# Alterations in Gut Microbiota and Their Correlation with Brain Beta-Amyloid Burden Measured by ^18^F-Florbetaben PET in Mild Cognitive Impairment Due to Alzheimer’s Disease

**DOI:** 10.3390/jcm13071944

**Published:** 2024-03-27

**Authors:** Geon Ha Kim, Bori R. Kim, Hai-Jeon Yoon, Jee Hyang Jeong

**Affiliations:** 1 Department of Neurology, Ewha Womans University College of Medicine, Seoul 07804, Republic of Korea; geonha@ewha.ac.kr; 2 Ewha Medical Research Institute, Ewha Womans University, Seoul 07804, Republic of Korea; borikim@ewha.ac.kr; 3 Department of Nuclear Medicine, Ewha Womans University College of Medicine, Seoul 07804, Republic of Korea

**Keywords:** mild cognitive impairment, amyloid beta, positron-emission tomography, amyloid, standardized uptake value ratio, gut microbiota

## Abstract

**(1) Background:** This study investigated changes in the gut microbial composition of individuals with mild cognitive impairment (MCI) due to Alzheimer’s disease (AD) and their relationship with positron emission tomography (PET) amyloid accumulation. **(2) Methods:** In total, 17 cognitively normal individuals without amyloid-beta (Aβ) accumulation (Aβ^−^NC) and 24 with Aβ-positive mild cognitive impairment (Aβ^+^MCI) who underwent ^18^F-florbetaben PET and fecal bacterial 16S ribosomal RNA gene sequencing were enrolled. The taxonomic compositions of the Aβ^−^NC and Aβ^+^MCI groups were compared. The abundance of taxa was correlated with the standardized uptake value ratio (SUVR), using generalized linear models. **(3) Results:** There were significant differences in microbiome richness (ACE, *p* = 0.034 and Chao1, *p* = 0.024), alpha diversity (Shannon, *p* = 0.039), and beta diversity (Bray–Curtis, *p* = 0.018 and Generalized UniFrac, *p* = 0.034) between the Aβ^−^NC and Aβ^+^MCI groups. The global SUVR was positively correlated with the genus Intestinibacter (*q* = 0.006) and negatively correlated with the genera Roseburia (*q* = 0.008) and Agathobaculum (*q* = 0.029). **(4) Conclusions:** In this study, we identified significant changes in the gut microbiota composition that occur in individuals with MCI due to AD. In particular, the correlation analysis results between PET amyloid burden and gut microbial abundance showed that amyloid deposition is associated with a reduction in specific taxa involved in butyrate production.

## 1. Introduction

Mild cognitive impairment (MCI) associated with Alzheimer’s disease (AD) represents a critical stage in the AD continuum and is characterized by cognitive deficits that exceed the expected age-related changes but do not meet the criteria for dementia [1]. Considering the suboptimal treatment options for advanced AD, early intervention during the MCI stage is potentially crucial to prevent or delay the onset of AD. With an estimated median time to progression to the mild dementia stage of 2 years [2], MCI due to AD has gained recognition as a significant public health concern, given its propensity to progress to more severe forms of dementia.

The complex relationship between AD and the gut microbiome, commonly known as the brain–gut axis, has emerged as a topic of increasing interest. Mounting evidence has supported the pivotal role of the gut microbiome in AD pathogenesis, particularly in the accumulation of amyloid beta (Aβ), a hallmark of the disease. In the context of gut dysbiosis, gut microbes have the potential to release amyloid-promoting factors that may escape from the gastrointestinal tract, penetrate the blood–brain barrier, and enter the central nervous system, potentially influencing the accumulation and absorption of Aβ42 [3].

Aβ deposition is a central pathological feature of AD, and its detection is essential for diagnosis and research. Techniques such as cerebrospinal fluid (CSF), positron emission tomography (PET) imaging, and blood-based biomarkers can be utilized to quantify Aβ. In particular, PET imaging provides in vivo visualization of Aβ deposition, enabling quantification of Aβ deposition at both the global and regional levels. Currently, three fluorine-18 amyloid PET tracers, ^18^F-florbetapir (Amyvid™), ^18^F-flutemetamol (Vizamyl™), and ^18^F-florbetaben (Neuraceq™), are approved for regular clinical application and have undergone validation in comparison to the Consortium to Establish a Registry for Alzheimer’s Disease (CERAD) pathology, which serves as the gold standard [4].

While a multitude of studies have reported alterations in the gut microbiome along the AD continuum, research investigating the correlation between PET amyloid burden and the gut microbiome remains scarce. Analyzing changes in gut microbial composition based on the accumulation of Aβ deposition in the brain measured from PET scans may offer novel and robust evidence supporting the hypothetical role of the gut–brain axis in AD pathophysiology. In this study, we compared the gut microbial composition between Aβ-negative cognitively normal controls (Aβ^−^NCs) and Aβ-positive individuals with MCI due to AD (Aβ^+^MCIs). Ultimately, we analyzed the relative abundance of the gut microbiota and its correlation with PET amyloid deposition at both the global and regional brain levels, aiming to identify microbial taxa demonstrating significant associations with PET-measured Aβ burden in the predementia stages.

## 2. Materials and Methods

### 2.1. Subjects

This study received approval from the institutional review boards (EUMC 2018-08-005-002 and 2020-09-006-002), with informed consent secured from all participants. This study was conducted in accordance with the principles of the 1975 Declaration of Helsinki (2013 version). We adhered to all applicable institutional and governmental regulations regarding the ethical use of human volunteers during the study. Between September 2018 and August 2021, 41 subjects meeting the specified inclusion criteria were recruited for this study: patients who (1) underwent ^18^F-FBB PET; (2) provided stool samples; (3) did not have a medication history of lipid-lowering drugs or probiotics, known to impact the gut microbiota, within four weeks of enrollment; and (4) were free from concurrent inflammatory bowel disease, infectious colitis, etc., and had not taken antibiotics within six weeks of enrollment. To eliminate the potential influence of different lifestyles (e.g., diet and exercise), ethnicities, and regions on the gut microbial composition, all participants recruited were community-dwelling Korean-nationality adults who were ordinary residents of Seoul. Clinical history, neurological examinations, laboratory findings, neuropsychological test results, and neuroimaging studies, including PET and MRI, were categorized into diagnostic groups. The ^18^F-FBB PET data were visually assessed by expert PET readers (Y.H.J. and K.J.Y.) without knowledge of any clinical information, and Aβ positivity was determined [5]. The criteria for diagnosing MCI adhered to the guidelines proposed by the NIA-AA [1]. In total, 17 of the 41 subjects were cognitively normal controls who were Aβ-negative (Aβ^−^NC), while 24 were Aβ-positive MCI (Aβ^+^MCI). 

### 2.2. ^18^F-FBB PET Imaging

^18^F-FBB was manufactured and handled according to good manufacturing practices at certified PET manufacturing sites and then delivered to our institutional PET imaging center. All ^18^F-FBB PET/CT scans were conducted following our institution’s established protocol, using a dedicated PET/CT scanner (Biograph mCT, Siemens Medical Solutions, Erlangen, Germany). Subjects received a single intravenous injection of 300 MBq 18F-FBB. A spiral CT image of the brain was acquired with CT parameters of 120 kV, 30 mAs, and a slice thickness of 1.0 mm. Brain PET images were captured in 3-dimensional mode from 90 to 110 min post-injection. The attenuation of the PET emission data obtained from the CT scans was corrected. To reduce motion artifacts, the subject’s head was secured using a head holder and fixation gear composed of a vacuum cushion. Standard PET data were reconstructed into a 128 × 128 matrix with a voxel size of 3.18 × 318 × 2.02 mm^3^, utilizing the built-in three-dimensional ordered subset expectation maximization (3D OSEM) algorithm, which included four iterations, 12 subsets, and a 5 mm Gaussian filter.

### 2.3. Image Analysis

For quantitative analysis, co-registration of ^18^F-FBB PET images to three-dimensional T1-weighted magnetic resonance (3D T1 MR) images was performed individually for each subject, using PMOD v4.0 (PMOD Technologies Ltd., Zurich, Switzerland). Volumes of interest (VOIs) were defined through an automated maximum probability atlas method, involving segmentation of each subject’s MRI into three probability maps (gray matter, white matter, and cerebrospinal fluid) and leveraging the Automated Anatomical Labeling (AAL) atlas within PMOD. Additionally, interframe motion correction was applied to the early dynamic images.

We calculated the regional standardized uptake value ratio (SUVR) as follows. First, the standardized uptake value (SUV) was determined for the volumes of interest (VOIs) in the cortical regions of the brain. According to amyloid PET staging [6,7], the precuneus and posterior cingulate exhibit the earliest signs of amyloid accumulation in Alzheimer’s disease (AD). Therefore, we separated the precuneus region from the parietal cortex and combined it with the posterior cingulate cortex to calculate the SUV. The temporal cortex was divided into medial and lateral regions, and the SUV was calculated for each region. Consequently, we obtained SUV values for the frontal, parietal (excluding the precuneus), precuneus/posterior cingulate, lateral and medial temporal, and occipital cortices. The SUV values of these distinct cortical regions were divided by the SUV of the reference region, which was the cerebellum (CBL), to calculate the regional SUVR. The global SUVR was defined as the non-weighted average of the values from all target regions.

### 2.4. Stool DNA Extraction and Bacterial 16S rRNA Gene Sequencing

Stool samples were frozen immediately after defecation at −20 °C and stored at −80 °C for 24 h. Stool DNA extraction was carried out within one month, using a standardized kit, which included a lysis buffer (CJ Bioscience, Seoul, Republic of Korea), following the manufacturer’s guidelines.

The V3–V4 hypervariable regions of the 16S rRNA gene were amplified using primers 341F and 805R through the direct PCR method. DNA libraries were then prepared using the NEBNext Ultra II FS DNA Library Prep Kit for Illumina (New England Biolabs Inc., Ipswich, MA, USA). The sequencing of these DNA libraries was carried out at CJ Bioscience Inc., using the Illumina MiSeq platform (Illumina, San Diego, CA, USA) with the 2 × 300 base-pair kits. 

### 2.5. 16S rRNA Gene-Based Microbiome Taxonomic Profiling and Analysis

Microbiome taxonomic profiling (MTP) was performed using the EZBioCloud platform (ChunLab Inc., Seoul, Republic of Korea) with the PKSSU4.0 database version [8]. The operational taxonomic unit (OUT) picking was conducted with UCLUST and CDHIT with a 97% similarity cutoff [9]. Comparisons of the taxonomic composition between the Aβ^−^NC and Aβ^+^MCI groups were performed using the comparative MTP analyzer implemented in EZBioCloud. Microbial richness and alpha diversity were analyzed and compared between the Aβ^−^NC and Aβ^+^MCI groups, using the Wilcoxon rank-sum test. Beta diversity was assessed and contrasted among these groups through pairwise Permutational Multivariate Analysis of Variance (PERMANOVA). These analyses were conducted using normalized data, considering variations in gene copy number. Microbial richness was measured by the ACE and Chao indices. Alpha diversity was measured by the Shannon and Simpson indices. Beta diversity was measured by Bray–Curtis and generalized UniFrac distances. The identification of taxonomic biomarkers with differential abundance between the two groups was conducted using the linear discriminant analysis effect size (LEfSe) algorithm [10]. Only taxa with LEfSe values > 3.0 are reported.

The abundance of taxa was correlated with global and regional SUVRs by generalized linear models implemented in Multivariate Association with Linear Models (MaAsLin) packages of RStudio (version 0.98.983) [11], adjusting for the effects of age and BMI (a confounding variable) in the study population. All analyses in MaAslin were performed using the default options. The resulting *p*-values were corrected for multiple comparisons at each phylogenetic level, using Benjamini–Hochberg correction (FDR). Statistical significance was defined as a *q*-value less than 0.05.

### 2.6. Statistical Analysis

Basic statistical analyses were conducted using two commercial software programs: IBM SPSS Statistics, version 26.0 (Armonk, NY, USA); and Rex 3.6.0 (Rexsoft, Seoul, Republic of Korea). The Shapiro–Wilk test was utilized to evaluate the normality of the data. Numerical data were presented as mean ± standard deviation (SD) when the normality test was passed, and as median with interquartile range (IQR) when the normality test was not passed. The differences in demographics and cognitive profiles between the Aβ^−^NC and Aβ^+^MCI groups were analyzed using Chi-squared or Fisher’s exact tests for categorical variables and Student’s *t*-test or the Mann–Whitney test for continuous variables, based on the normality test outcomes. Similarly, differences in SUVR values between the groups were assessed using Student’s *t*-test or the Mann–Whitney test, depending on the normality test results. All statistical tests were two-sided, with a significance threshold set at *p* < 0.05.

## 3. Results

### 3.1. Patient Characteristics

Table 1 provides a summary of the baseline characteristics, cognitive scores, and global SUVRs of the 41 subjects. Overall, there were no significant differences in baseline features between the two groups, except for BMI and APOE4 positivity. The Aβ^+^MCI group exhibited a significantly lower BMI than the Aβ^-^NC group, and the proportion of APOE4-positive individuals was significantly greater in the Aβ^+^MCI group. Cognitive scores were also found to be significantly different between the groups. MMSE scores were significantly lower, while global CDR scores were significantly greater in the Aβ^+^MCI group than in the Aβ^−^NC group. Additionally, the global and regional SUVRs were significantly greater in the Aβ^+^MCI group than in the Aβ^−^NC group.

### 3.2. Richness and Diversity of the Gut Microbiota

**Figure 1:**
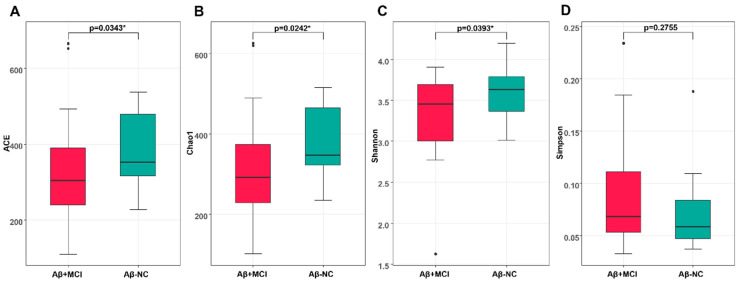
The box plots depict the differences in the alpha diversity between the Aβ^−^NC and Aβ^+^MCI groups using the (**A**) ACE, (**B**) Chao 1, (**C**) Shannon, and (**D**) Simpson indices. * *p* < 0.05.

We compared the microbiome richness and diversity between the Aβ^−^NC and Aβ^+^MCI groups (Figure 1). The microbial richness of stool swabs evaluated at the species level was significantly lower in the Aβ^+^MCI group than in the Aβ^−^NC group, as measured by ACE and Chao1 (*p* = 0.034 and *p* = 0.024, respectively).

Statistically significant differences were observed in the alpha diversity of gut microbial taxa between the Aβ^−^NC and Aβ^+^MCI groups, as determined by the Shannon index (*p* = 0.039). However, no statistically significant differences were found based on the Simpson index (*p* = 0.276). 

**Figure 2:**
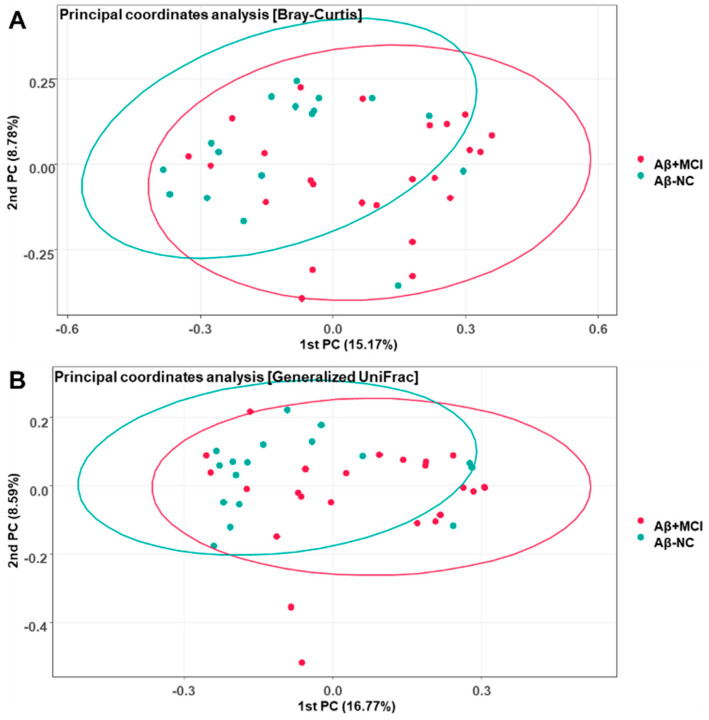
Principal coordinate analysis plots depicting the differences in beta diversity between the Aβ^−^NC and Aβ^+^MCI groups based on (**A**) Bray–Curtis dissimilarity (PERMANOVA, F = 1.1761, *p* = 0.018) and (**B**) Generalized UniFrac distance (PERMANOVA, F = 1.667, *p* = 0.034). The first and second principal coordinates (PCs) are displayed on the *x*-axis and *y*-axis, respectively, and the numbers in parentheses indicate the percentage of community variation explained.

The Bray–Curtis and Generalized UniFrac principal coordinate analysis (PCoA) plots of the Aβ^−^NC and Aβ^+^MCI samples demonstrated variations (Figure 2). In the Bray–Curtis PCoA plot of Figure 2A, the first and second principal coordinate axes (1st PC and 2nd PC) explain 15.17% and 8.78% of the community variation at the species level, respectively. Meanwhile, in the Generalized UniFrac plot of Figure 2B, the first and second principal coordinate axes (1st PC and 2nd PC) explain 16.77% and 8.59% of the community variation at the species level, respectively. PERMANOVA also suggested that there were significant differences between the Aβ^−^NC and Aβ^+^MCI samples (F = 1.1761, *p* = 0.018 for Bray–Curtis; F = 1.667, *p* = 0.034 for Generalized UniFrac).

### 3.3. Alterations in the Gut Microbial Composition in Aβ^+^MCI Patients

**Figure 3:**
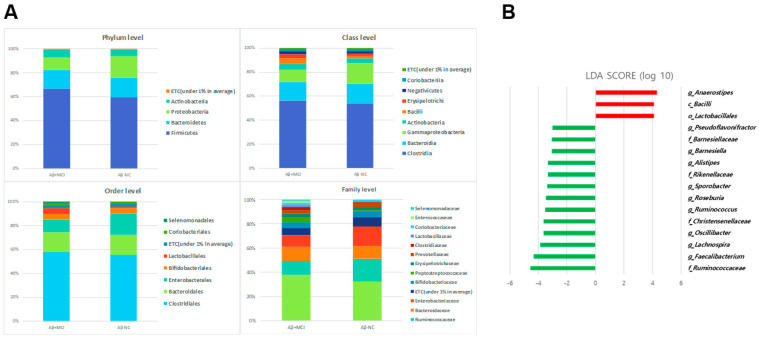
(**A**) The gut microbial composition of the Aβ^−^NC and Aβ^+^MCI groups is illustrated, showing the bacterial communities present in both groups across different taxonomic levels. Bar graphs represent the relative abundance of taxa at the phylum, class, order, and family levels. (**B**) The LEfSe analysis between the Aβ^−^NC and Aβ^+^MCI groups are depicted in the histogram of LDA scores for differentially abundant taxa. Positive LDA scores, shown in red, indicate an enrichment of taxa in the Aβ^+^MCI group, while negative LDA scores, displayed in green, suggest an enrichment of taxa in the Aβ^−^NC group. The taxa with LDA scores (log10) > 3 are listed, differentiating the microbial profiles between the two groups.

The comparisons of gut microbial compositions between the Aβ^−^NC and Aβ^+^MCI groups are shown in Figure 3A at various taxonomic levels. Predominantly, the groups shared Firmicutes, Bacteroidetes, and Proteobacteria at the phylum level, with Actinobacteria also being present. At the class level, *Clostridia*, *Bacteroidia*, *Gammaproteobacteria*, *Actinobacteria*, *Bacilli*, *Erysipelotrichi*, *Negativicutes*, and *Coriobacteriia* emerged as the leading bacteria. The order level highlighted five major bacteria: *Clostridiales*, *Bacteroidales*, *Enterobacteriales*, *Bifidobacteriales*, and *Lactobacillales*. Dominant bacteria at the family level included *Lachnospiraceae*, *Ruminococcaceae*, *Bacteroidaceae*, and *Enterobacteriaceae*. Overall, the gut microbial composition in the Aβ^+^MCI group closely aligned with that of the Aβ^−^NC group.

However, distinct differences in microbiota between the Aβ+MCI patients were unveiled using the LEfSe analysis. Specifically, an increase in the relative abundances of the class *Bacilli* (*p* = 0.034), order *Lactobacillales* (*p* = 0.047), and genus *Anaerostipes* (*p* = 0.036) was noted in the Aβ^+^MCI group. In contrast, the abundances of the families *Ruminococcaceae* (*p* = 0.006), *Christensenellaceae* (*p* = 0.002), *Rikenellaceae* (*p* = 0.011), and *Barnesiellaceae* (*p* = 0.015); and the genera *Faecalibacterium* (*p* = 0.014), *Lachnospira* (*p* = 0.008), *Oscillibacter* (*p* = 0.009), *Ruminococcus* (*p* = 0.034), *Roseburia* (*p* = 0.039), *Sporobacter* (*p* = 0.022), *Alistipes* (*p* = 0.011), *Barnesiella* (*p* = 0.013), and *Pseudoflavonifractor* (*p* = 0.014) were significantly diminished (LDA score cutoff > 3.0, Figure 3B).

### 3.4. Associations between the Gut Microbial Taxa Composition and Brain Aβ Burden

**Figure 4:**
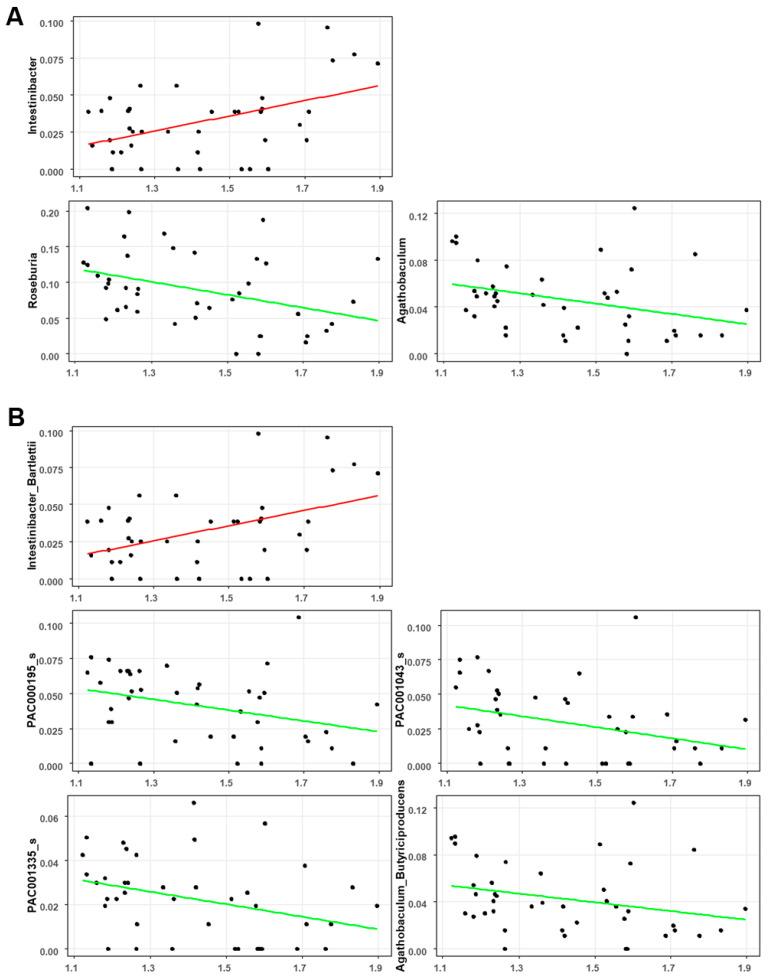
Significant correlation between the global SUVR and gut microbiota composition at the (**A**) genus level and (**B**) species level.

The correlation between the global brain Aβ burden and gut microbial abundance was analyzed after adjusting for patient age and BMI. At the genus level, the global SUV was positively correlated with *Intestinibacter* (*CE* = 1.27; *p* = 0.001; *q* = 0.006; Figure 4A) and negatively correlated with *Roseburia* (*CE* = −1.38; *p* = 0.002; *q* = 0.008; Figure 4A) and *Agathobaculum* (*CE* = −1.20; *p* = 0.008; *q* = 0.029; Figure 4A). At the species level, the global SUV was positively correlated with *Intestinibacter Bartlettii* (*CE* = 1.27; *p* = 0.001; *q* = 0.006; Figure 4B) but negatively correlated with *PAC000195* (*CE* = −1.05; *p* = 0.012; *q* = 0.042; Figure 4B), *PAC001043* (*CE* = −1.18; *p* = 0.011; *q* = 0.041; Figure 4B), *PAC001335* (*CE* = −0.94; *p* = 0.012; *q* = 0.044; Figure 4B), and *Agathobaculum butyriciproducens* (*CE* = −1.22; *p* = 0.006; *q* = 0.023; Figure 4B). 

**Figure 5:**
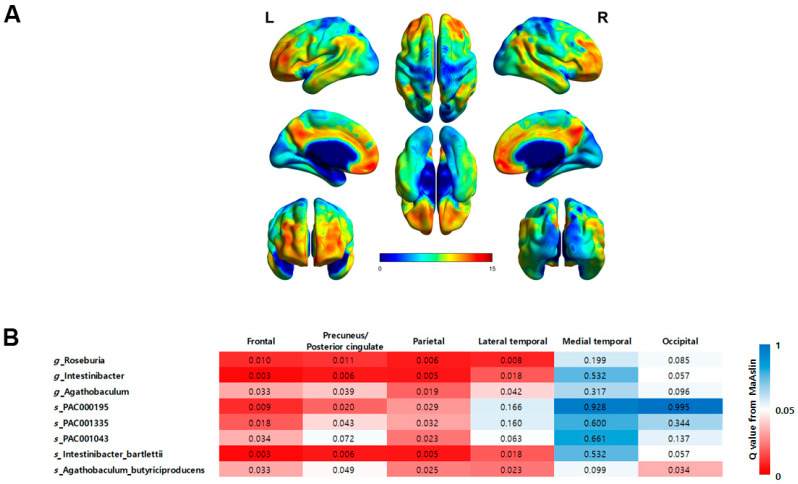
(**A**) Statistical parametric maps obtained from FBB images between the Aβ^−^NC and Aβ^+^MCI groups demonstrate an increase in amyloid deposition in typical AD regions, especially the frontal cortex, precuneus/posterior cingulate cortex, parietal cortex, and lateral temporal cortex (FWE-corrected *p* < 0.05, *t* > 5.17). (**B**) A heatmap illustrating the significance of correlations between regional brain Aβ burden and the eight taxa that displayed significant correlations with the global brain Aβ burden. The significance levels are represented by *q*-values, with darker shades of red indicating a greater degree of significance (*q* < 0.05).

The correlation between regional brain Aβ burden and eight taxa that showed a significant correlation with global brain Aβ burden was analyzed after adjusting for patient age and BMI (Figure 5). Consistent with the global SUVRs, the regional SUVRs in the frontal and lateral parietal cortex were significantly correlated with all eight taxa. For the precuneus/posterior cingulate cortex, the regional SUVRs showed a significant correlation with all taxa except for the species *PAC001043*. In the lateral temporal cortex, the regional SUVRs were significantly correlated with *Roseburia*, *Intestinibacter*, and *Agathobaculum* at the genus level and with *Intestinibacter bartlettii* and *Agathobaculum butyriciproducens* at the species level. In the occipital cortex, only the species *Agathobaculum butyriciproducens* showed a significant correlation. In the medial temporal cortex, no taxa showed a significant correlation.

## 4. Discussion

This study compared the gut microbial composition between Aβ^−^NC and Aβ^+^MCI patients. The microbial richness, as gauged by the ACE and Chao1 indices, was found to be significantly lower in the Aβ^+^MCI group compared to the Aβ^−^NC group. Similarly, alpha diversity, assessed using the Shannon index, was also significantly reduced in the Aβ^+^MCI group relative to the Aβ^−^NC group. However, when evaluating alpha diversity with the Simpson index, no statistically significant difference was observed between the two groups. Decreased richness and diversity have been reported in various pathological conditions, including neurodegenerative disorders such as AD and Parkinson’s disease [12,13,14]. The significant difference in the Shannon index between groups, with no significant difference observed in the Simpson index, can be interpreted as being driven by the notable alterations in the overall richness and distribution of various microbial species in the Aβ^+^MCI group compared to the Aβ^−^NC group, despite the lack of substantial differences in the dominance of specific species between the groups. This finding implies that changes in the gut microbiota composition in individuals with Aβ^+^MCI may be more closely associated with alterations in the overall community structure rather than with the dominance of specific species. The significant variation in beta diversity, as determined by the Bray–Curtis and generalized UniFrac principal coordinate analysis plots, between the Aβ^−^NC and Aβ^+^MCI groups indicates substantial dissimilarities in the overall microbial community structure between individuals without Aβ accumulation and those with Aβ associated with MCI. 

According to the results of the LEfSe analysis, the relative abundance of the class *Bacilli* and order *Lactobacillales* increased in the Aβ^+^MCI group. Zhuang et al. also reported an increase in *Actinobacteria* and *Bacilli* at the class level and an increase in *Lactobacillales* at the order level in AD patients compared to those in the control group [15]. In our study, we observed a decrease in *Ruminococcaceae*, *Christensenellaceae*, *Rikenellaceae*, and *Barnesiellaceae* at the family level. *Ruminococcaceae* has been widely reported to play a beneficial role in the maintenance of gut health by producing butyrate and other short-chain fatty acids (SCFAs), and most studies have reported a decrease in abundance in individuals with AD [12,14,16,17]. *Christensenellaceae* is also known to play a crucial role in gut health, acting as a keystone species in the development of the symbiotic gut microbiome. Research has shown that individuals diagnosed with Crohn’s disease (CD) or ulcerative colitis (UC) demonstrate significantly lower levels of Christensenellaceae in their gut microbiota. Liu et al. reported a decrease in *Christensenellaceae* abundance in patients with depression [18]. Nagpal et al. reported an increase in *Christensenellaceae* due to a modified Mediterranean–ketogenic diet, which is known to potentially contribute to a reduction in AD-related pathologies [19]. A systematic review and meta-analysis reported a reduced abundance of Rikenellaceae in patients with AD spectrum as compared to healthy controls [20]. Although changes in *Barnesiellaceae* in the gut microbiome of individuals with AD have not been reported, one study indicated a negative correlation between *Barnesiellaceae* taxonomic units and pulse wave velocity (PWV) [21]. PWV increases alongside arterial stiffness (AS), which has been associated with AD due to its impact on cerebral vasculature. Therefore, the negative correlation between PWV and *Barnesiellaceae* abundance implies a potential decrease in *Barnesiellaceae* abundance in the disease group. In this study, we observed a decrease in the abundance of the genera *Faecalibacterium* [22], *Lachnospira* [23], *Oscillibacter* [24], *Ruminococcus* [25], *Roseburia* [26,27], and *Alistipes* [28] in the Aβ^+^MCI patients, and this finding is consistent with the findings of previous studies in MCI and AD patients. In contrast, our findings of a reduction in the abundance of the genera *Sporobacter* and *Alistipes* in Aβ^+^MCI patients differed from previous findings. Lee et al. reported a negative correlation between cognitive function and the population of *Sporobacter*, and Jo et al. additionally reported higher LDA scores for *Sporobacter* in Parkinson’s disease patients, implying a predominant result of increased *Sporobacter* in disease cohorts; however, this finding was limited in AD research [29,30].

The main advantage of this study is the use of amyloid PET scans for the in vivo visualization of Aβ deposition. Additionally, PET imaging enables the quantification of Aβ deposition at both the global and regional levels. In this study, we analyzed the relative abundance of the gut microbiota and its correlation with PET-derived amyloid deposition at both the global and regional brain levels, aiming to identify microbial taxa demonstrating significant associations with PET-measured Aβ burden. The global SUVR was positively correlated with the genus *Intestinibacter*. The genus *Intestinibacter* contains only one species, *Intestinibacter Bartlettii* (reclassified from the genus *Clostridium* in 2014 [31]), which also shows a significant positive correlation with the global SUVR. In a limited number of studies, *Intestinibacter bartlettii* has been reported to be a potentially harmful bacterium in neurodevelopmental disorders and moyamoya disease [32,33]. A consistent finding in gut microbial changes induced by metformin was a decrease in the abundance of *Intestinibacter Bartlettii* [34,35]. Several clinical studies have reported a decrease in dementia risk and improvements in memory and executive function with metformin [36]. 

The negative correlation between the global SUVR and the genera *Roseburia* and *Agathobaculum* is particularly noteworthy. Such a negative correlation between the global SUVR and the genus *Agathobaculum* seems to be attributed to *Agathobaculum butyriciproducens* at the species level. *Agathobaculum butyriciproducens* is a butyrate-producing bacterium that plays a crucial role in maintaining a balanced gut microbiota, regulating the host immune response, and enhancing intestinal mucosal barrier function [37,38]. A preclinical study revealed that the administration of *Agathobaculum butyriciproducens* improved cognitive function and AD pathology [37]. The genus *Roseburia*, known for producing butyrate, is recognized as a protective bacterium in AD, and its abundance decreased in the AD group [26,27,39]. The remaining *PAC000195*, *PAC001043*, and *PAC001335*, which were negatively correlated with global SUVRs, belong to the family *Lachnospiraceae*, and the beneficial effects of butyrate-producing *Lachnospiraceae* have been reported in dementia [40].

The concept of a “leaky gut”, marked by heightened intestinal permeability as a result of gut microbiome dysbiosis, facilitates the translocation of microbiome-derived compounds into the systemic circulation. This process is linked to the onset of AD hallmarks, which encompass an escalation in amyloid load, the emergence of amyloid plaques, the development of neurofibrillary tangles, prolonged neuroinflammation, and the activation of microglial cells and astrocytes. These processes ultimately lead to neuronal loss, neurodegeneration, and cognitive impairment, culminating in AD [3]. The SUVR is the most widely used quantitative indicator of brain Aβ deposition [4]. In this study, we identified significant negative correlations between the global SUVR and the abundance of butyrate-producing bacteria. Maintaining an adequate level of butyric acid production in the gut lumen helps in balancing the gut microbiota, regulating the host immune response, and enhancing intestinal mucosal barrier function. Butyrate-producing bacteria ferment undigested carbohydrates in the intestinal lumen, resulting in the production of acidifying SCFAs such as butyric acid. Consequently, promoting the growth of butyrate-producing bacteria as probiotics may be beneficial for gut health [38]. In AD as well, the recovery or promotion of the growth of butyrate-producing bacteria as probiotics is expected to have protective effects, such as delaying the onset of AD. Butyrate-producing bacteria play a crucial role as key modulators of intestinal permeability and have shown a positive effect on decreasing Aβ fibrillation in preclinical models [41,42], and further research is needed to confirm this in the clinical setting. Our findings indicate that a reduction in butyrate-producing bacteria is associated with Aβ deposition in the brain. However, even if a negative correlation is observed between the overall SUVR and specific butyrate-producing bacteria, this can only prove correlation and cannot explain causality due to upstream and downstream relationships without further study.

Aβ deposition can also be measured in plasma, but the particular advantage of PET is its ability to assess Aβ deposition regionally. We further analyzed the correlation between regional brain Aβ burden and nine taxa that exhibited a significant correlation with global brain Aβ burden. The analysis revealed that the frontal and parietal cortex exhibited the most taxa with a significant correlation, whereas the medial temporal and occipital cortex did not show significant correlations for most taxa. This may be because the subjects included in this study were in the MCI stage, which is a stage before full-blown AD. According to the process of Aβ accumulation in AD, it first invades the frontal and parietal nerves and then spreads to the remaining associative neocortex [43]. The level of taxa showing significant correlations with regional SUVRs appears to reflect PET staging, but this requires further study.

In this study, the Aβ-negative cohort comprised subjects who abstained from lipid-lowering drugs or probiotics for four weeks prior to enrollment, as these can affect gut microbiota. Additionally, they had no concurrent inflammatory bowel disease, infectious colitis, or recent antibiotic use. Neurological examinations, laboratory findings, neuropsychological tests, and neuroimaging studies were normal, and PET scans showed no Aβ deposition. Participants were community-dwelling Korean adults residing in Seoul to mitigate the impact of diverse lifestyles, ethnic backgrounds, and geographical locations on gut microbial compositions. However, controlling for the effects of diet, exercise, external stressors, and immune function on gut microbiota remains challenging and is a major drawback of this study. As is often observed in carefully controlled and well-designed studies, participant selection bias, variances in sample size, sequencing methods, bioinformatics processing, and confounding variables may also influence the outcome [44]. Therefore, standardized clinical trials will be necessary in the future to comprehensively unravel the complex interactions within the microbiota–gut–brain axis involved in AD. 

Another limitation of this study is that we did not measure the butyric acid levels of the subjects. An additional study verifying the reduction of butyric acid levels in fecal samples is needed to support a role for butyrate-producing bacteria in brain Aβ deposition. Lastly, the utilization of bacterial 16S rRNA gene sequencing may have limitations in providing comprehensive and detailed information to establish gut microbial alterations. Recent technological advancements have introduced advanced options such as long-read sequencing or whole-genome sequencing. 

## 5. Conclusions

We identified significant changes in the gut microbiota composition that occur in individuals with MCI due to AD. In particular, the correlation analysis results between PET amyloid burden and gut microbial abundance showed that amyloid deposition is associated with a reduction in specific taxa involved in butyrate production. Butyrate-producing bacteria play a crucial role in maintaining a balanced gut microbiota, regulating the host immune response, and enhancing intestinal mucosal barrier function. The results of this study support the hypothetical role of the gut–brain axis in AD pathophysiology by confirming a significant correlation between specific taxa involved in maintaining gut mucosal barrier function and quantitative parameters of Aβ deposition measured via in vivo brain PET.

## Figures and Tables

**Table 1:** General features and cognitive scores of subjects.

	Total	Aβ^−^NC (*n* = 17)	Aβ^+^MCI (*n* = 24)	*p*-Value
Age	72.12 ± 7.02	70.82 ± 7.63	73.04 ± 6.56	0.3391
Sex, female	22 (53.66%)	10 (58.82%)	12 (50%)	0.8101
HTN	17 (41.46%)	6 (35.29%)	11 (45.83%)	0.724
DM	12 (29.27%)	6 (35.29%)	6 (25%)	0.5072
Hyperlipidemia	16 (39.02%)	8 (47.06%)	8 (33.33%)	0.5737
Smoking	8 (19.51%)	2 (11.76%)	6 (25%)	0.2618
Alcohol	5 (12.2%)	3 (17.65%)	2 (8.33%)	0.6327
BMI	23.01 (22, 24.9)	24.14 (23.01, 26.3)	22.69 (21.29, 23.72)	0.0209 *
Right-handed	39 (95.12%)	16 (94.12%)	23 (95.83%)	>0.99
APOE4 carrier	15 (36.59%)	1 (5.88%)	14 (58.33%)	0.0019 *
Education, years	12 (6, 14)	12 (6, 16)	12 (9, 12.5)	0.538
MMSE score	27 (25, 29)	29 (28, 30)	25 (22, 26)	<0.001 *
Global CDR	0.5 (0.5, 0.5)	0.5 (0, 0.5)	0.5 (0.5, 0.5)	0.0015 *
Global SUVR	1.43 ± 0.23	1.2 ± 0.05	1.6 ± 0.17	<0.001 *
Regional SUVR				
Frontal	1.49 ± 0.27	1.23 ± 0.06	1.68 ± 0.19	<0.001 *
Precuneus/ Posterior cingulate	1.49 ± 0.29	1.2 ± 0.06	1.7 ± 0.19	<0.001 *
Parietal	1.42 ± 0.23	1.2 ± 0.07	1.57 ± 0.17	<0.001 *
Lateral temporal	1.37 ± 0.24	1.14 ± 0.05	1.54 ± 0.19	<0.001 *
Medial temporal	1.17 ± 0.09	1.13 ± 0.08	1.2 ± 0.1	0.017 *
Occipital	1.39 ± 0.2	1.23 ± 0.04	1.5 ± 0.18	<0.001 *

This table was summarized as appropriate according to a normality test and the presence of warning from a chi-squared test. Values are reported as mean with standard deviation or median with interquartile range. * *p*-values are significant at the 0.05 level. Abbreviations: Aβ^−^NC, Aβ-negative cognitively normal control; Aβ^+^MCI, Aβ-positive mild cognitive impairment; DM, diabetes mellitus; HTN, hypertension; APOE, apolipoprotein E; MMSE, Mini-Mental State Examination; CDR, Clinical Dementia Rating; SUVR, standardized uptake value ratio.

## Data Availability

The datasets used and analyzed in the current study are available from the corresponding author upon reasonable request.
